# 

**Published:** 1915-04

**Authors:** 


### ANNOUNCEMENTS.

NORTHERN OHIO DENTAL ASSOCIATION.—The annual meeting of this society will be held this year in Cleveland on June 3, 4 and 5. This is one of the oldest societies in the country and as it always has an enthusiastic meeting no one can miss its meetings without serious loss to himself.

DR. W. A. PRICE, *President.*
C. D. PECK, *Secretary.*

---

MICHIGAN STATE BOARD OF DENTAL EXAMINERS.—The next regular meeting of the Michigan State Board of Dental Examiners, for the examination of applicants who wish to practice dentistry in Michigan, will be held in the Dental College at Ann Arbor, beginning Monday, June 14, 1915, at 8:00 A. M., and continue through Saturday, June 19.

For application blanks and full information apply to

A. W. HAIDLE, *Secretary,*
Negaunee, Michigan.

---

INDIANA STATE DENTAL ASSOCIATION.—Will hold its annual meeting this year in Indianapolis, at the Claypool Hotel, May 18, 19, 20. It is an Indiana program but visitors from other states will be welcome.

---

KENTUCKY DENTAL ASSOCIATION.—Will meet this year in Ashland, June 8th to 10th. This is a new place of meeting and ought to bring out all the eastern Kentucky, West Virginia and southern Ohio dentists.

---

THE TENNESSEE STATE ASSOCIATION.—Will meet this year in Sewanee, the home of J. P. Corley. It ought to be a bright and enthusiastic meeting.

